# Developmental changes in the categorical processing of positive and negative facial expressions

**DOI:** 10.1371/journal.pone.0201521

**Published:** 2018-08-03

**Authors:** Michael Vesker, Daniela Bahn, Franziska Degé, Christina Kauschke, Gudrun Schwarzer

**Affiliations:** 1 Department of Developmental Psychology, Justus-Liebig-Universität Gießen, Giessen, Germany; 2 Clinical Linguistics, Department of German Linguistics, Philipps-Universität Marburg, Marburg, Germany; University of Colorado Boulder, UNITED STATES

## Abstract

Categorical biases in the processing of emotional facial expression have been the subject of much debate in the literature. Opposing views on this topic claim either that positive or negative facial expressions enjoy improved processing in the human brain. The developmental changes in the processing advantages of positive and negative facial expressions are also disputed, with studies using varying paradigms showing seemingly contradictory results. Therefore, to further investigate the development of categorical processing and extraction of emotional information from faces, we tested 6-, 9-, and 12-year-old children, as well as adults, on their ability to categorize various facial expressions as positive or negative as quickly as possible. This was a simplified paradigm designed to explicitly contrast the processing efficiency of positive and negative facial expressions on the broader level of those emotional valence categories, rather than specific single emotional expressions. Our results show an early age processing advantage for positive facial expressions, which disappears in adults who show no such differences in the case of response time measures. In the case of accuracy measures, the early advantage for positive facial expressions gradually disappears and is reversed into a negativity advantage in adults. These findings demonstrate that category-based positive and negative processing advantages are strongly modulated by age over the course of development, and can exhibit opposite effects depending on the developmental stage of the participant.

## Introduction

The rapid and accurate recognition of emotional facial expressions is a crucial component of social interaction. This is true for both ordinary social interactions among friends and family, and for situations which call for a rapid response in order to ensure one’s own survival; such as reading anger in an approaching rival’s face, or fear in the face of another person who has detected a source of danger. The initial recognition of facial expressions as belonging to the broad pleasant/positive or unpleasant/negative emotion categories in terms of valence is a very basic form of processing emotional information from faces, which according to ERP measurements, occurs earlier than the recognition of specific expressions (see [1] for a review). In fact, the ability to recognize specific emotional expressions is thought to originate from an initial sorting of all facial expressions into positive and negative categories, which eventually develops into the fully mature ability to identify individual emotional expressions as children grow up [2,3]. Developmental changes in the usage of these broad emotion categories and possible advantages for processing emotional information from faces belonging to either category are the focus of much discussion in the literature, with various studies occasionally showing somewhat contradictory results. On the one hand, there are studies showing a bias favoring the processing of positive facial expressions in children and adults, particularly when it comes to the identification or labeling of specific emotions expressed in the faces (e.g., De Sonneville et al., 2002) [4]. On the other hand, when children and adults were asked only to detect the presence of a particular facial expression (rather than to identify expressions from a number of possible choices), negative faces showed a detection advantage across all age groups [5]. It was therefore our goal in the present study to further examine possible developmental changes in processing advantages linked to the positive and negative emotion categories of facial expressions with a focus on the explicit usage of those categories. Therefore, we chose a task which only required a broad positive/negative emotional categorization of facial expressions, rather than identifying or detecting specific emotional expressions. Using this task, we investigated the speed and accuracy of categorizing different facial expressions as positive or negative in 6, 9, and 12-year old children compared to adults.

The role played by positive and negative emotional categories in the perception of facial expressions has also been extensively studied in adults with mixed, sometimes contradictory results. Some researchers have found that negative facial expressions enjoy improved processing over positive ones [6–11]. However, other researchers have shown results which demonstrate that it is positive (i.e. happy) faces which enjoy superior processing [12–17], and the truth of the matter has yet to be fully resolved.

Despite the enormous amount of attention paid to this matter in adulthood, the question of how the processing advantages associated with these broad emotion categories might shift over the course of development has not been studied as extensively thus far beyond infancy. Infant studies have shown that during the first year of life, infants undergo significant shifts in their attentional biases. Studies have shown newborns, 4-month-olds, and even 6-month-olds preferring to look at happy faces [18,19], after which point they begin to show attentional preferences towards negative faces between the ages of 5 and 8 months, with the exact time point of this transition remaining a matter of some debate (see [20] for a review). For instance, a study by Heck and colleagues [21] showed that while at 3.5 months infants did not display significant differences in looking preference towards either happy or fearful faces versus a checkerboard, 5-month-old infants showed a greater preference for looking at fearful faces as compared to happy faces. In another study [22], 5-month-old infants did not show looking preferences between happy and fearful faces, while infants tested at 7 months showed a preference for fearful faces.

Developmental studies which examined the processing of positive and negative emotional faces in older children have also shown some disagreements in their findings. Although these studies concentrated on testing the perception and processing of individual emotions rather than whole categories, there can be said to be a prevalence of results showing a bias favoring the perception of positive faces, particularly when it comes to the identification or labeling of specific emotional expressions. For instance, De Sonneville and colleagues (2002) [4] showed photos of happy, sad, angry, and afraid faces to children ranging from 7 to 10 years of age, as well as adults, and found that all subjects were faster and more accurate at identifying pairs of faces as showing the same emotion when that emotion was happiness compared to all the other emotions. The authors of another study [23] showed happy, sad, angry, afraid, and disgusted faces to 5-, 7-, and 10-year-old children and adults at varying intensities, and found that happy faces allowed for the best performance even at lower intensities, especially in the youngest children. In another study [24], the authors presented children ranging from 5 to 17 years of age and adults with photos of happy, sad, angry, afraid, disgusted and surprised faces with varying levels of visual noise, and children showed the best performance (lower signal strength threshold for correct identification) with happy faces. Mancini, Agnoli, Baldaro, Bitti, & Surcinelli (2013) [25], investigated the changes in accuracy for the emotional identification of happy, sad, angry, afraid and disgusted faces by children ranging from 8 to 11 years of age, and found the highest accuracy for identifying happy faces. Thus, tasks requiring the specific identification of emotional facial expressions seem to be prone to showing an advantage for identifying positive faces. This advantage for identifying positive faces over negative faces in children could be caused by an inherent advantage of identifying positive expressions due to the natural structure of this face category: There is less ambiguity in identifying positive emotions when the only such primary expression is happiness, in contrast to the greater variety of primary negative emotions, i.e. sadness, anger, fear, and disgust [26].

However, when LoBue (2009) [5] presented 5-year-old children and adults with a task which required only the detection of a particular facial expression rather than its identification, a bias favoring the detection of negative faces was observed in both 5-year-old children and adults. Fearful, angry, and sad faces were detected faster compared to happy ones in a series of experiments where subjects needed to find the target emotion from an array which also contained the opposite valence distractor (e.g. finding a happy face from an array with 1 happy face and 8 angry distractor faces). Thus, tasks requiring the detection of a specific emotional face from an array of distractors seem to show better detection of negative faces in children as well as adults [5]. This advantage for detecting negative over positive facial expressions could be explained by a higher visual sensitivity towards threat-relevant stimuli and/or inherent differences in the visual saliency of negative and positive expressions which might not necessarily be reflected in efficiency of processing the emotional information they contain (see also Vaish, Grossman, & Woodward (2008) [20] for an overview on the negativity bias).

Thus, when it comes to the question of the development of children’s ability to process different facial expressions, the results are somewhat mixed, which might at least to some degree depend on whether participants are required to retrieve specific emotion labels, and how directly the broad positive and negative emotion categories are utilized in the experimental task. In other words, it would seem that happy faces enjoy processing advantages when subjects are asked to identify the specific emotion on display, while various negative expressions such as fear or anger enjoy processing advantages when the task at hand instead requires the detection of the task relevant face, a conclusion also reached by Nummenmaa and Calvo (2015) [27] in a review of the topic. It was therefore the aim of the present study to examine the development of the proposed processing advantage of positive or negative emotional expressions using a task which would require the explicit positive/negative categorization of facial expressions, rather than the identification or detection of specific emotional facial expressions. Such a basic task of sorting facial expressions into a positive or negative category allowed us to directly assess the processing of the broad positive and negative categories of facial expressions. We accomplished this by asking children and adults to categorize different emotional expressions as positive or negative as quickly as possible. This task had the advantages of being simple enough that even the youngest children in our study could understand it as well as any adult, and would not require the retrieval of specific emotion labels while still requiring participants to process the emotional information contained in the faces.

The only study we are aware of which used a similar methodology was that of Tottenham, Phuong, Flannery, Gabard-Durnam, & Goff (2013) [28]. This study was, however, focused on the categorization of emotionally ambiguous stimuli such as surprised and neutral faces, which, from their own results are not truly perceived as neutral in terms of emotional valance. Instead, their results show that neutral and surprised faces are more often perceived as negative, especially by younger children. For this reason, we chose to avoid using neutral and surprised faces in the present study, and showed only clearly positive (different types of happy and happy-surprised) and clearly negative emotional expressions (angry, fearful, sad). We also avoided the use of disgusted faces since prior research shows that children under the age of 9 (which we intended to examine in the present study), are not yet able to properly understand disgusted expressions [29,30]. Using this approach, we tried to balance our selection of facial expressions as evenly as possible across the positive and negative categories, in order to ensure that children and adults would not be biased by the overall proportions of positive and negative faces.

In our study, we tested children from three age groups (6-, 9-, and 12-years-old) as well as adults. We chose these age groups because they reflect the typical bounds of starting primary school where children undergo a period of intense socialization, and the transition to adolescence at which point children begin to strongly resemble adults in their face-processing capabilities [24]. These groups also fit the age range from 5 to 12 years old, during which children show significant improvements in the processing of emotional facial expressions [24] and emotion words [31–33]. The group of adult participants additionally allowed us to compare the developmental course of emotion-categorization proficiency in childhood to the ability of adults in the same task.

## Materials and methods

### Participants

For our study we tested 82 children and 24 adults. The children were organized in 3 age groups (6-, 9-, and 12-year old) while the adults made up our 4th age group. The children were either recruited through a contact database at the University of Giessen, or through primary schools in the cities of Giessen and Marburg in the German state of Hessen. All children performed two pre-tests before experimental data-collection to verify normal development: A German vocabulary test, “Wortschatz- und Wortfindungstest” (WWT 6–10) [34], and a non-verbal pattern-matching intelligence test, Raven’s Colored Progressive Matrices (CPM). Of the 82 child participants tested in total, 10 were excluded due to abnormally low scores on the pretests, leaving 72 participants (24 per age group, with half being female) whose data was analyzed in the present study. All children were German-born, native German speakers, and of Caucasian background.

The adult group consisted of 24 Caucasian undergraduate students (12 females) from the faculty of Psychology and Sports Science at the University of Giessen, and were recruited through a university mailing list.

### Stimuli

The stimuli used for the present study comprised of 48 color photographs of 8 adult Caucasian models (4 male, and 4 female, all having some theatrical experience) producing 24 positive and 24 negative facial expressions. All photographs were taken from the database of the Pell laboratory at McGill University [35], and were verified to be recognizable as their intended expression by an emotion-identification survey conducted at the Pell lab. This survey offered 8 choices of emotions per facial expression, and the results indicated a high average correct recognition rate of 90.8% for the faces chosen as stimuli in the present study.

**Fig 1:**
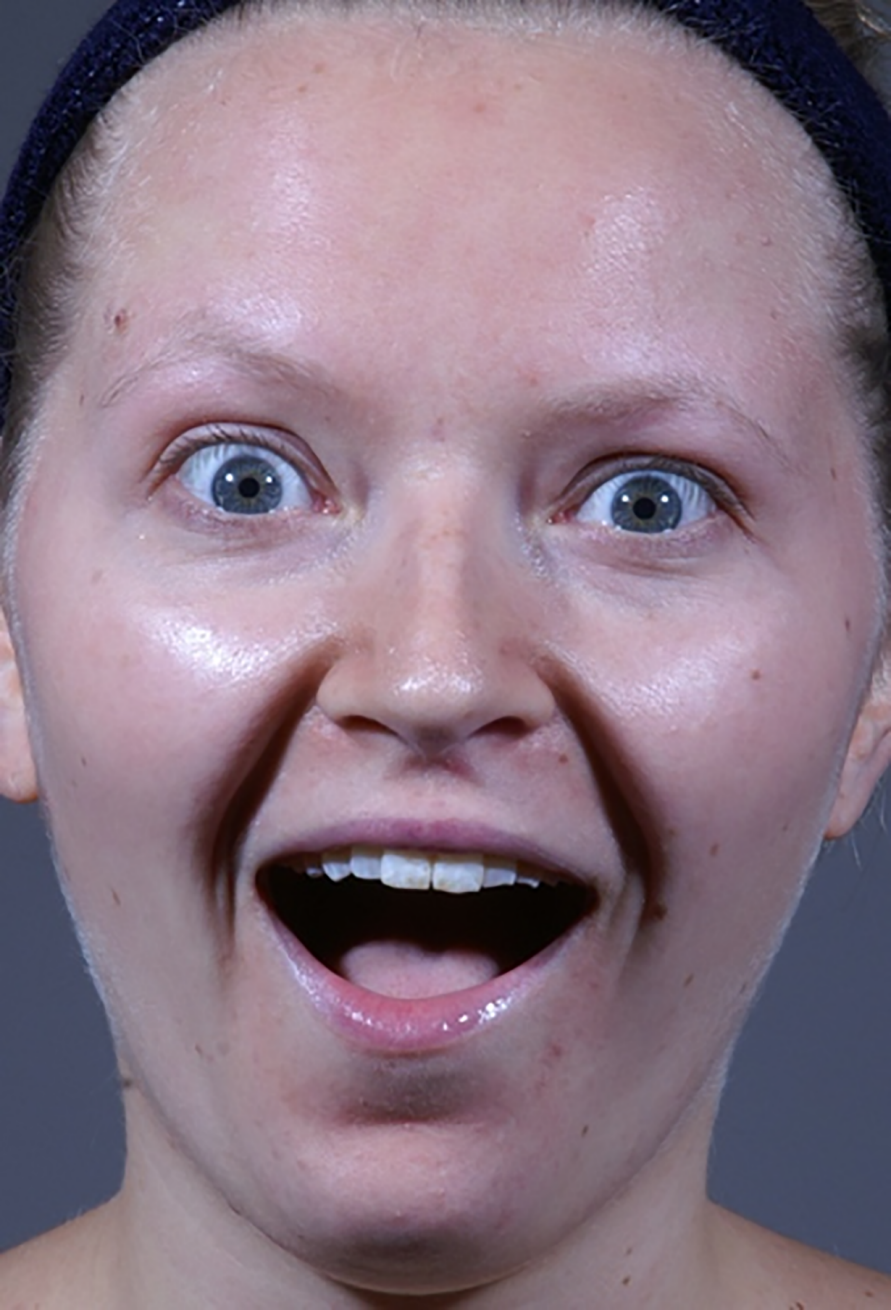
Happy-surprised example. Example of a positive happy-surprised stimulus face used in the present study [35].

Each model appeared in 6 of the photographs used in our experiment (3 positive and 3 negative). The 24 positive photographs comprised of 12 happy faces with a closed mouth, 5 happy faces with a slightly opened mouth, and 7 happy-surprised faces with a widely opened mouth (see Fig 1 for an example of a happy-surprised face). The 24 negative photographs comprised of 6 angry faces, 6 fearful faces, and 12 sad faces.

This selection of facial expressions for the positive and negative emotion categories was a result of striving to make sure that each category is composed of a variety of expressions, in order to truly represent a category of positive and negative emotional expressions, rather than merely contrasting happy faces against the negative expressions. We also balanced the two categories in terms of mean arousal and valence using ratings by adult subjects in a previous study [36] using a 7-point SAM valence scale [37], and a 5-point SAM arousal scale [37]. This meant that the arousal across the categories was approximately equal, and that the positive category mean should be as positive as the negative category is negative, i.e. that the mean valence values of the two categories are be equidistant from the value corresponding to a neutral valence. Thus, by ensuring that on the one hand our positive and negative face categories each contained a variety of expressions, while on the other hand still maintaining parity in terms of the overall average arousal and valence, we aimed to focus the study on the categories themselves, rather than the individual emotions contained within.

### Apparatus and procedure

The present study was conducted in accordance to the German Psychological Society (DGPs) Research Ethics Guidelines. The Office of Research Ethics at the University of Giessen approved the experimental procedure and the informed consent protocol. Written informed consents were obtained from the children’s parents prior to their participation in the study.

Children in the 6-year-old and 9-year-old groups were tested in two sessions, with the first session used to conduct the WWT and CPM as pre-tests, followed by the experimental data collection in the second testing session. Children in the 12-year-old group went through the pre-tests and the experiments in a single session. Adults only performed the experiment itself, also in a single testing session.

During the experiment, participants first performed a practice block of 12 trials where they classified 6 positive and 6 negative African and Asian faces as either positive or negative emotionally. If a participant made no more than a single error out of the 12 trials, the main experimental block would begin. If a participant made more than one error during the practice, the practice block would repeat for a second time, before proceeding to the experimental block regardless of error rate (this was the case for 4 6-year-olds, 4 9-year-olds, 5 12-year-olds, and 3 adults.). The experimental block consisted of 48 trials where each of the 48 facial stimuli described above appeared once, in a randomized order, and participants were asked to classify them as positive or negative as rapidly as possible. Each trial began with the appearance of a facial stimulus, which would remain on the screen until the participant pressed one of two designated buttons to indicate their classification choice, at which time the photograph would disappear, and the screen would remain black for 1000 milliseconds before the start of the next trial.

Responses were made using the two mouse buttons on a laptop with a 15.6 inch screen using OpenSesame 2.9 [38] for stimulus presentation control. The laptop keyboard was covered with a removable stencil, leaving only the task-relevant buttons available to press. The stencil also had a stylized symbol next to each of the buttons, corresponding to the button’s assigned classification choice: A raincloud for negative, and a sun for positive category selection. Whether each button corresponded to a positive or a negative classification was assigned randomly, and the experimenter would begin each testing session by affixing the stencil with the right symbol orientation to the keyboard.

Upon completing the testing sessions, each child participant in the 6- and 9-year-old age groups received a choice of a small toy as a present (for a total of 2 presents over the course of the experiment), while the 12-year old children were offered either a choice of 2 presents at once, or a single cinema voucher at the end of their single session. The adult participants received either course-credit or 5 Euros as a reward for their participation, selected by each participant individually.

## Results

### Accuracy

Accuracy measures on a per-trial basis were analyzed with a two-way ANOVA, using the stimulus emotion-category as a within-subject factor, and age-group as a between-subjects factor.

**Fig 2:**
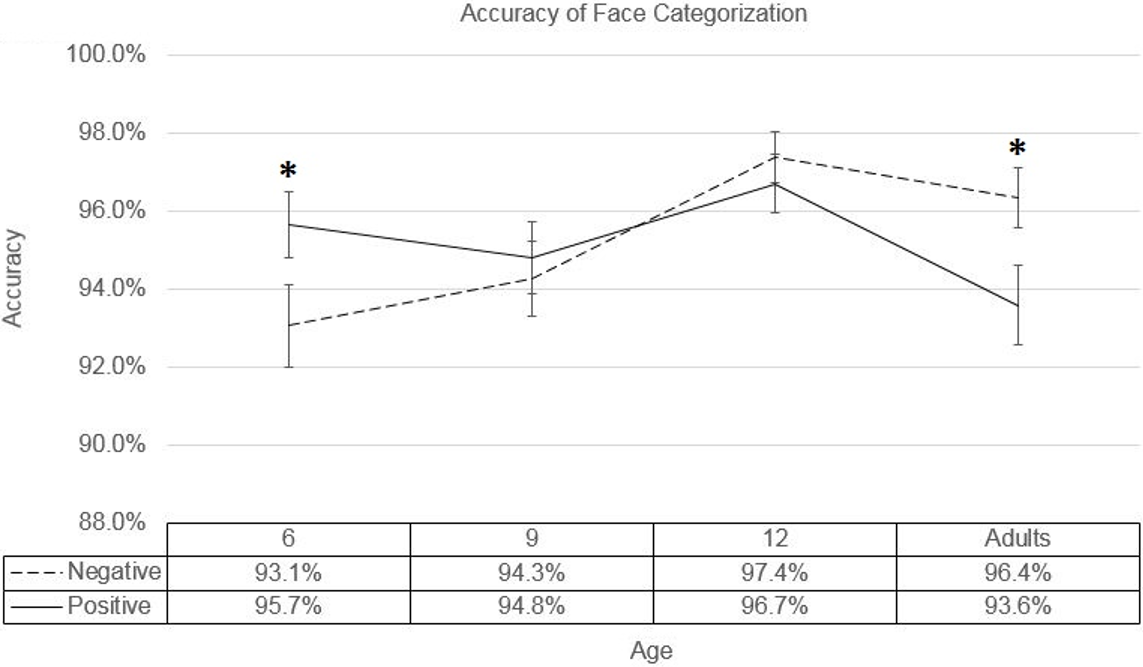
Measures of accuracy. Accuracy rates for categorization of positive and negative faces over all age groups. Stars indicate the significance level of differences between positive and negative faces within each corresponding age group. Error bars represent standard Error.

The analysis detected a significant effect of age (*F*(3,4600) = 3.919, *p =* .008, partial *η*^*2*^ = .003), and a significant interaction between age and emotion-category (*F*(3,4600) = 3.223, *p =* .022, partial *η*^*2*^ = .002). While older age groups generally showed a higher degree of accuracy overall, pair-wise post-hoc tests carried out on the interaction of age and emotion-category revealed that while the 9- and 12-year-old groups did not show significantly different accuracy rates between positive and negative faces (*p =* .678 and *p =* .580, respectively), 6-year old children displayed significantly higher accuracy for positive faces (*p =* .038, Cohen’s *d =* 0.113), while adults showed significantly higher accuracy for negative faces (*p =* .027, Cohen’s *d =* 0.128) (see the means and standard errors in Fig 2).

### Response time

An ANOVA analysis was also carried out on the response time measures of correct key-presses on a per-trial basis. As above, emotion category was used as a within-subject factor, and age-group as a between-subjects factor. For this analysis we eliminated any outlier responses below 100ms or above 2 standard deviations over the mean response time (calculated individually for each participant).

The analysis showed significant main effects of age (*F*(3,4164) = 475.39, *p <* .001, partial *η*^*2*^ = .255), and emotion-category (*F*(1,4164) = 53.175, *p <* .001, partial *η*^*2*^ = .013), as well as a significant interaction between these two factors (*F*(3,4164) = 3.128, *p =* .025, partial *η*^*2*^ = .002).

The main effect of age manifested itself as a gradual decrease in response time with increasing age, with a post-hoc Tuckey’s HSD test showing differences between all age groups to be significant (*p <* .001, Cohen’s *d* = 0.788 for the difference between 6-year-olds and 9-year olds; *p <* .001, Cohen’s *d* = 0.797 for the difference between 12-year-olds and adults) with the exception of the difference between the 9- and 12-year-old age groups (*p =* .459), showing a plateau in development between these two stages.

**Fig 3:**
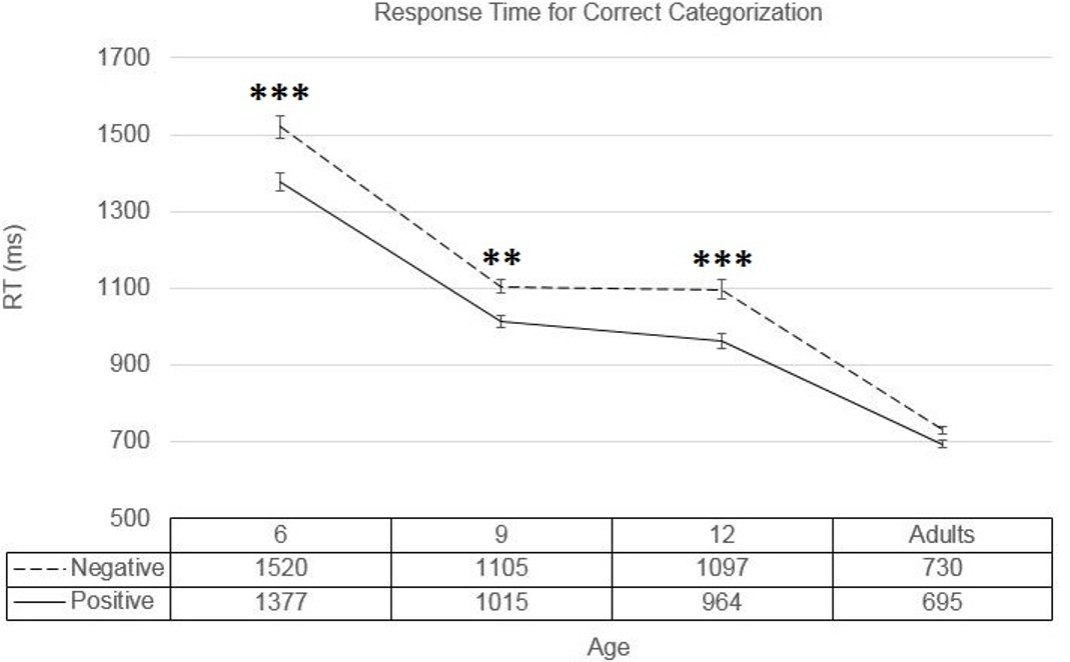
Measures of correct response times. Response times for correct categorization trials for positive and negative faces for all age groups. Stars indicate the significance level of differences between positive and negative faces within each corresponding age group. Error bars represent standard error.

The main effect of emotion-category showed an overall faster response speed for positive faces relative to negative ones. Post-hoc comparisons between these two categories within each age group were carried out to examine the interaction between age and emotion-category, and showed the difference between positive and negative faces to be significant in all the children’s age groups (*p <* .001, Cohen’s *d =* 0.246; *p =* .001, Cohen’s *d =* 0.240; and *p <* .001, Cohen’s *d =* 0.258 for the 6, 9, and 12 year old groups, respectively). However, the adults showed no significant difference between positive and negative faces (*p =* .194) (see means and standard errors in Fig 3).

## Discussion

In the present study we investigated the development of biases in the processing of positive and negative emotional expressions on the level of the broader positive and negative emotional categories, rather than the specific emotion contained within. We therefore used a task which explicitly required children and adults to respond to our stimuli on the level of those categories, and didn’t require the retrieval of specific emotion labels, or the detection of specific emotional expressions.

Our results show that in terms of response speed, children initially show a “positivity bias”, correctly categorizing positive faces faster than negative ones. However, this advantage erodes with age until no significant differences can be observed between the categorization speed of positive and negative faces in the adult age group. This result fits the findings of two earlier studies which found that children perceive positive faces as more arousing than adults, while negative faces are perceived very similarly in this regard [36,39]. This could at least partially account for the faster categorization of positive faces by children, as arousal is known to modulate visual attention, and potentially processing speed [40,41]. These results also corroborate the positivity advantage found in children’s face processing by some researchers [4,23–25], while disagreeing with the “negativity bias” found in children by others [5]. Our results regarding response speed also agree with those of another study [42], which examined valence categorization with stimuli from another modality, namely, the categorization of emotion words in children and adults. The results of this study showed that positive emotion words also enjoyed a processing speed advantage over negative emotion words, but only up to the age of 6 years, after which point the difference in the speed of accurately categorizing emotion words as positive or negative was no longer significant.

In terms of accuracy, our findings demonstrated opposite effects of valence-category depending on the age group examined. The youngest age group of 6-year-old children displayed a positivity advantage in their accuracy, more accurately categorizing positive faces than negative faces, i.e. more often incorrectly pressing the positive response button for negative faces than vice versa. Meanwhile, the oldest age group of adults displayed the opposite effect of a negativity advantage, more accurately categorizing negative faces than positive faces, i.e. more often incorrectly pressing the negative response button for positive faces than vice versa. The age groups of 9- and 12-year-old children did not themselves display any significant differences in categorization accuracy between positive and negative facial expressions, but they did show a gradual transition from the positivity bias of the 6-year-old children to the negativity bias of the adult age group. Thus, the accuracy of processing the broad categories of positive and negative emotional expressions changed from an advantage for processing positive emotional expressions in young children to an advantage for processing negative emotional expressions in adults.

We therefore see that even simple emotion categorization is highly influenced by age. Looking at both our response time and accuracy measures, we see an overall tendency towards a positivity biases at an early age, which disappears with increasing age. However, while the increased speed of correctly categorizing positive faces relative to negative ones in children could at least to some degree be explained by the increased perceived arousal of positive faces for children as compared to adults, the same cannot be easily said for the results of the accuracy measures. This raises the question of why young children tend to make more errors when categorizing negative faces, while adults make more errors when categorizing positive faces? Although the absolute magnitude of these differences was relatively low, the fact that they appeared even in such an easy task makes it interesting to examine the cause of this developmental reversal of response biases.

Such a tendency could be explained in terms of the societal conditions and social-emotional needs of children as compared to adults, i.e. the social relevance of the facial expressions in question. While the traditional argument of those who support the view of negative emotion processing advantages is that negative emotions carry more survival-relevant information [7,43–47], we speculate that this might be most applicable to situations encountered by adults, as they would have a great physical capacity to respond to potential threats individualistically compared to children. Conversely, children might benefit more from seeking the protection of an adult, which may be facilitated by the positivity bias we found in the younger age groups.

This age-dependent shift in the relative importance of processing positive versus negative stimuli is also reflected in changes that occur as adults age. Several studies have shown that when compared to younger adults (in their mid-20’s), older adults (over 50 years of age) display positivity biases just as the younger children do in our own study. For instance, [48] found that while younger adults did not show any differences between positive and negative faces, older adults were able to respond faster to positive faces as well as being able to more accurately recall them afterward. A separate study [17] found that older adults showed accuracy deficits compared to younger adults in identifying negative, but not positive faces. Thus, we can see that younger adults, who are best able to effectively act on negative information through their high capacity for physical action tend to show the so-called negativity bias. On the other hand, age groups with a lower capacity for physical action such as children and older adults tend to display a positivity bias. This fits the argument of evolutionary relevance, as older adults and children may be better off seeking the protection and help of others rather than confronting dangerous situations independently [36,39].

### Conclusions

Our results regarding the sorting of facial expressions into the broad categories of positive and negative emotions generally show an early positivity bias that gradually disappears with increasing age. Response time data indicate an equalization of processing for positive and negative faces by the stage of adulthood, while accuracy data show a reversal of the early positivity bias into a negativity bias in adults. These results demonstrate that both positivity and negativity biases undergo gradual shifts over the course of development. These shifts might occur as a result of changes in the social-emotional relevance of the information carried by facial expressions, or as a function of a person’s exposure to various facial expressions (or quite possibly some combination of both).

### Limitations

Our study was limited by the omission of age ranges which cover the later stages of adolescence, which could be highly interesting as adolescents undergo a number of shifts in their societal roles and interactions at that stage of development. Likewise, we also did not examine the possible effects of age-related decline in emotion categorization by studying older adults. The experiment was also limited in the usage of adult faces as stimuli, and given our hypothesis that social relevance might play an important role in forming categorical processing biases, it would be very interesting to see how participants, especially children respond to children’s faces which can carry very different social implications to children and adults. Finally, it would be useful to conduct future studies with a greater number of models posing for stimuli to increase the generalizability of the results.

## References

[1] Calvo MG, Nummenmaa L. Perceptual and affective mechanisms in facial expression recognition: An integrative review. Cogn Emot [Internet]. 2016;30(6):1081–106. Available from: 10.1080/02699931.2015.1049124 26212348

[2] Widen SC. Children’s Interpretation of Facial Expressions: The Long Path from Valence-Based to Specific Discrete Categories. Emot Rev [Internet]. 2013 1 [cited 2017 Jan 27];5(1):72–7. Available from: http://journals.sagepub.com/doi/10.1177/1754073912451492

[3] Widen SC, Russell JA. Differentiation in Preschooler’s Categories of Emotion. Emotion. 2010;10(5):651–61. 10.1037/a0019005 21038948

[4] De Sonneville LMJ, Verschoor CA, Njiokiktjien C, Op het Veld V, Toorenaar N, Vranken M. Facial Identity and Facial Emotions: Speed, Accuracy, and Processing Strategies in Children and Adults. J Clin Exp Neuropsychol [Internet]. 2002;24(2):200–13. Available from: http://www.tandfonline.com/doi/abs/10.1076/jcen.24.2.200.989 10.1076/jcen.24.2.200.989 11992203

[5] Lobue V. More than just another face in the crowd: Superior detection of threatening facial expressions in children and adults. Dev Sci. 2009;12(2):305–13. 10.1111/j.1467-7687.2008.00767.x 19143803

[6] Eastwood JD, Smilek D, Merikle PM. Differential attentional guidance by unattended faces expressing positive and negative emotion. Percept Psychophys [Internet]. 2001;63(6):1004–13. Available from: http://www.ncbi.nlm.nih.gov/pubmed/11578045 11578045 10.3758/bf03194519

[7] Eastwood JD, Smilek D, Merikle PM. Negative facial expression captures attention and disrupts performance. Percept Psychophys [Internet]. 2003;65(3):352–8. Available from: http://www.ncbi.nlm.nih.gov/pubmed/12785065 12785065 10.3758/bf03194566

[8] Fox E, Lester V, Russo R, Bowles RJ, Pichler A, Dutton K. Facial Expressions of Emotion: Are Angry Faces Detected More Efficiently? Cogn Emot [Internet]. 2000;14(1):61–92. Available from: http://www.tandfonline.com/doi/abs/10.1080/026999300378996 10.1080/026999300378996 17401453 PMC1839771

[9] Horstmann G, Bauland A. Search asymmetries with real faces: testing the anger-superiority effect. Emotion. 2006;6(2):193–207. 10.1037/1528-3542.6.2.193 16768552

[10] Lobue V, Deloache JS. Superior detection of threat-relevant stimuli in infancy. Dev Sci. 2010;13(1):221–8. 10.1111/j.1467-7687.2009.00872.x 20121878

[11] Pinkham AE, Griffin M, Baron R, Sasson NJ, Gur RC. The face in the crowd effect: anger superiority when using real faces and multiple identities. Emotion. 2010;10(1):141–6. 10.1037/a0017387 20141311

[12] Calvo MG, Beltran D. Recognition advantage of happy faces: Tracing the neurocognitive processes. Neuropsychologia. 2013;51(11):2051–60. 10.1016/j.neuropsychologia.2013.07.010 23880097

[13] Johnson KJ, Fredrickson BL. ‘“We All Look the Same to Me”‘ Positive Emotions Eliminate the Own-Race Bias in Face Recognition. Psychol Sci. 2005;16(11):875–81. 10.1111/j.1467-9280.2005.01631.x 16262774 PMC1808554

[14] Juth P, Lundqvist D, Karlsson A, Öhman A. Looking for Foes and Friends: Perceptual and Emotional Factors When Finding a Face in the Crowd. Emotion [Internet]. 2005;5(4):379–95. Available from: http://doi.apa.org/getdoi.cfm?doi=10.1037/1528-3542.5.4.379 10.1037/1528-3542.5.4.379 16366743

[15] Kirita T, Endo M. Happy face advantage in recognizing facial expressions. Acta Psychol (Amst). 1995;89:149–63.

[16] Leppänen JM, Hietanen JK. Positive facial expressions are recognized faster than negative facial expressions, but why? Psychol Res [Internet]. 2004 12 29 [cited 2015 Sep 25];69(1–2):22–9. Available from: http://link.springer.com/10.1007/s00426-003-0157-2 10.1007/s00426-003-0157-2 14648224

[17] Sullivan S, Ruffman T, Hutton SB. Age differences in emotion recognition skills and the visual scanning of emotion faces. J Gerontol B Psychol Sci Soc Sci. 2007;62(1):P53–60. 17284558 10.1093/geronb/62.1.p53

[18] LaBarbera JD, Izard CE, Vietze P, Parisi SA. Four- and six-month-old infants’ visual responses to joy, anger, and neutral expressions. Child Dev [Internet]. 1976 6 [cited 2018 Jan 19];47(2):535–8. Available from: http://www.ncbi.nlm.nih.gov/pubmed/1269322 1269322

[19] Farroni T, Menon E, Rigato S, Johnson MH. The perception of facial expressions in newborns. Eur J Dev Psychol [Internet]. 2007;4(1):2–13. Available from: http://www.tandfonline.com/doi/abs/10.1080/17405620601046832 10.1080/17405620601046832 20228970 PMC2836746

[20] Vaish A, Grossman T, Woodward A. Not all emotions are created equal: The negativity bias in social-emotional development. Psychol Bull. 2008;134(3):383–403. 10.1037/0033-2909.134.3.383 18444702 PMC3652533

[21] Heck A, Hock A, White H, Jubran R, Bhatt RS. The development of attention to dynamic facial emotions. J Exp Child Psychol. 2016;147:100–10. 10.1016/j.jecp.2016.03.005 27064842 PMC5191507

[22] Peltola MJ, Leppänen JM, Mäki S, Hietanen JK. Emergence of enhanced attention to fearful faces between 5 and 7 months of age. Soc Cogn Affect Neurosci. 2009;4(2):134–42. 10.1093/scan/nsn046 19174536 PMC2686224

[23] Gao X, Maurer D. A happy story: Developmental changes in children’s sensitivity to facial expressions of varying intensities. J Exp Child Psychol [Internet]. 2010;107(2):67–86. Available from: http://linkinghub.elsevier.com/retrieve/pii/S0022096510000810 10.1016/j.jecp.2010.05.003 20542282

[24] Rodger H, Vizioli L, Ouyang X, Caldara R. Mapping the development of facial expression recognition. Dev Sci [Internet]. 2015 11 [cited 2015 Oct 20];18(6):926–39. Available from: http://www.ncbi.nlm.nih.gov/pubmed/25704672 10.1111/desc.12281 25704672

[25] Mancini G, Agnoli S, Baldaro B, Bitti PER, Surcinelli P. Facial expressions of emotions: recognition accuracy and affective reactions during late childhood. J Psychol [Internet]. 2013 1 20 [cited 2015 Oct 29];147(6):599–617. Available from: http://www.tandfonline.com/doi/abs/10.1080/00223980.2012.727891 10.1080/00223980.2012.727891 24199514

[26] Feyereisen P, Malet C, Martin Y. Is the faster processing of expressions of happiness modality-specific? Aspects of Face Processing. 1986.

[27] Nummenmaa L, Calvo MG. Dissociation between recognition and detection advantage for facial expressions: A meta-analysis. Emotion. 2015;15(2):243–56. 10.1037/emo0000042 25706834

[28] Tottenham N, Phuong J, Flannery J, Gabard-Durnam L, Goff B. A negativity bias for ambiguous facial-expression valence during childhood: converging evidence from behavior and facial corrugator muscle responses. Emotion [Internet]. 2013 2 [cited 2017 Jan 28];13(1):92–103. Available from: http://www.ncbi.nlm.nih.gov/pubmed/22906084 10.1037/a0029431 22906084 PMC4006094

[29] Widen SC, Russell JA. The “Disgust Face” Conveys Anger to Children. 2010;10(4):455–66. 10.1037/a0019151 20677863

[30] Widen SC, Russell JA. A Closer Look at Preschoolers ‘ Freely Produced Labels for Facial Expressions. Dev Psychol. 2003;39(1):114–28. 12518813 10.1037//0012-1649.39.1.114

[31] Baron-Cohen S, Golan O, Wheelwright S, Granader Y, Hill J. Emotion word comprehension from 4 to 16 years old: a developmental survey. Front Evol Neurosci [Internet]. 2010 [cited 2017 Jul 28];2:109 Available from: http://www.ncbi.nlm.nih.gov/pubmed/21151378 10.3389/fnevo.2010.00109 21151378 PMC2996255

[32] Kauschke C, Nutsch C, Schrauf J. Verarbeitung von konkreten und abstrakten Wörtern bei Kindern im Schulalter. Z Entwicklungspsychol Padagog Psychol [Internet]. 2012 1 17 [cited 2017 Jul 28];44(1):2–11. Available from: http://econtent.hogrefe.com/doi/abs/10.1026/0049-8637/a000045

[33] Schwanenflugel PJ, Akin CE. Developmental Trends in Lexical Decisions for Abstract and Concrete Words. Read Res Q [Internet]. 1994 [cited 2017 Jul 28];29(3):250–64. Available from: http://www.jstor.org/stable/747876

[34] Glück CW. Wortschatz- und Wortfindungstest für 6– bis 10–Jährige: WWT 6–10 Munich: Urban & Fischer/ Elsevier; 2011.

[35] Pell MD. Nonverbal emotion priming: Evidence from the “Facial Affect Decision Task.” J Nonverbal Behav. 2005;29(1):45–73.

[36] Vesker M, Bahn D, Degé F, Kauschke C, Schwarzer G. Perceiving arousal and valence in facial expressions: Differences between children and adults. Eur J Dev Psychol [Internet]. 2017;5629(February):1–15. Available from: https://www.tandfonline.com/doi/full/10.1080/17405629.2017.1287073

[37] Bradley MM, Lang PJ. MEASURING EMOTION: THE SELF-ASSESSMENT SEMANTIC DIFFERENTIAL MANIKIN AND THE SEMANTIC DIFFERENTIAL. J Behav Ther Exp Psychiat. 1994;25(1):49–59. 10.1016/0005-7916(94)90063-9 7962581

[38] Mathôt S, Schreij D, Theeuwes J. OpenSesame: an open-source, graphical experiment builder for the social sciences. Behav Res Methods [Internet]. 2012 6 [cited 2015 Jan 26];44(2):314–24. Available from: http://www.pubmedcentral.nih.gov/articlerender.fcgi?artid=3356517&tool=pmcentrez&rendertype=abstract 10.3758/s13428-011-0168-7 22083660 PMC3356517

[39] Picardo R, Baron AS, Anderson AK, Todd RM, Denham S, Zoller D, et al Tuning to the Positive: Age-Related Differences in Subjective Perception of Facial Emotion. McCormick CM, editor. PLoS One [Internet]. 2016 1 6 [cited 2017 Jan 26];11(1):e0145643 Available from: http://dx.plos.org/10.1371/journal.pone.0145643 10.1371/journal.pone.0145643 26734940 PMC4703339

[40] Schimmack U. Attentional Interference Effects of Emotional Pictures: Threat, Negativity, or Arousal? Emotion [Internet]. 2005;5(1):55–66. Available from: http://doi.apa.org/getdoi.cfm?doi=10.1037/1528-3542.5.1.55 10.1037/1528-3542.5.1.55 15755219

[41] Vogt J, De Houwer J, Koster EHW, Van Damme S, Crombez G. Allocation of spatial attention to emotional stimuli depends upon arousal and not valence. Emotion [Internet]. 2008;8(6):880–5. Available from: http://doi.apa.org/getdoi.cfm?doi=10.1037/a0013981 10.1037/a0013981 19102600

[42] Bahn D, Vesker M, García Alanis JC, Schwarzer G, Kauschke C. Age-Dependent Positivity-Bias in Children’s Processing of Emotion Terms. Front Psychol [Internet]. 2017 7 26 [cited 2017 Jul 28];8:1268 Available from: http://journal.frontiersin.org/article/10.3389/fpsyg.2017.01268/full 10.3389/fpsyg.2017.01268 28798706 PMC5526962

[43] Fox E, Lester V, Russo R, Bowles RJ, Pichler A, Dutton K. Facial Expressions of Emotion: Are Angry Faces Detected More Efficiently? Cogn Emot [Internet]. 2000;14(1):61–92. Available from: http://www.ncbi.nlm.nih.gov/pubmed/17401453 10.1080/026999300378996 17401453 PMC1839771

[44] Hansen CH, Hansen RD. Finding the face in the crowd: An anger superiority effect. J Pers Soc Psychol [Internet]. 1988 [cited 2016 Nov 26];54(6):917–24. Available from: http://doi.apa.org/getdoi.cfm?doi=10.1037/0022-3514.54.6.917 3397866 10.1037//0022-3514.54.6.917

[45] Öhman A, Lundqvist D, Esteves F. The face in the crowd revisited: A threat advantage with schematic stimuli. J Pers Soc Psychol. 2001;80:381–96. 11300573 10.1037/0022-3514.80.3.381

[46] Vuilleumier P, Armony JL, Driver J, Dolan RJ. Effects of attention and emotion on face processing in the human brain: an event-related fMRI study. Neuron. 2001;30(3):829–41. 11430815 10.1016/s0896-6273(01)00328-2

[47] Baumeister RF, Bratslavsky E, Finkenauer C, Vohs KD. Bad is stronger than good. Rev Gen Psychol [Internet]. 2001 [cited 2017 Jan 27];5(4):323–70. Available from: http://doi.apa.org/getdoi.cfm?doi=10.1037/1089-2680.5.4.323

[48] Mather M, Carstensen LL. AGING AND ATTENTIONAL BIASES FOR EMOTIONAL FACES. Psychol Sci. 2003;14(5):409–15. 10.1111/1467-9280.01455 12930469

